# PathFams: statistical detection of pathogen-associated protein domains

**DOI:** 10.1186/s12864-021-07982-8

**Published:** 2021-09-14

**Authors:** Briallen Lobb, Benjamin Jean-Marie Tremblay, Gabriel Moreno-Hagelsieb, Andrew C. Doxey

**Affiliations:** 1 Department of Biology, University of Waterloo, Waterloo, Ontario Canada; 2 Department of Biology, Wilfrid Laurier University, Waterloo, Ontario Canada

**Keywords:** Proteins of unknown function, Hypothetical proteins, Virulence factors, Pathogens, Environmental association, Lineage specificity

## Abstract

**Background:**

A substantial fraction of genes identified within bacterial genomes encode proteins of unknown function. Identifying which of these proteins represent potential virulence factors, and mapping their key virulence determinants, is a challenging but important goal.

**Results:**

To facilitate virulence factor discovery, we performed a comprehensive analysis of 17,929 protein domain families within the Pfam database, and scored them based on their overrepresentation in pathogenic versus non-pathogenic species, taxonomic distribution, relative abundance in metagenomic datasets, and other factors.

**Conclusions:**

We identify pathogen-associated domain families, candidate virulence factors in the human gut, and eukaryotic-like mimicry domains with likely roles in virulence. Furthermore, we provide an interactive database called PathFams to allow users to explore pathogen-associated domains as well as identify pathogen-associated domains and domain architectures in user-uploaded sequences of interest. PathFams is freely available at https://pathfams.uwaterloo.ca.

**Supplementary Information:**

The online version contains supplementary material available at 10.1186/s12864-021-07982-8.

## Background

Bacterial virulence factors are proteins that facilitate pathogen adherence, colonization, and survival with the host. Despite a long history of virulence factor characterization, new virulence factors and mechanisms are continually being discovered, even in well characterized organisms. Given the rapidly growing availability of genomic sequences across the bacterial tree of life, there is a potential explosion of virulence protein diversity to be discovered in genomes [1].

A key question in the bioinformatic identification and analysis of virulence factors is how to detect candidate virulence related proteins from sequence information. Common strategies include the use of online virulence factor databases such as the VFDB, which is a comprehensive, curated resource of virulence factors across the best-characterized bacterial pathogens [2] Protein sequences from newly sequenced organisms or metagenomes can be compared against the VFDB to identify homologs of known virulence factors, which include toxins and adherence factors as well as more general protein families that contribute, but are not specific, to virulence (e.g., flagellar proteins).

Although virulence factors can be grouped into protein sequence families, a more fundamental level of analysis is to assess proteins at the level of domains. Even if most of a protein sequence might be unrecognizable, the identification of key domains within that sequence may be sufficient to identify it as a candidate virulence factor [3]. Domains are modular units of proteins that adopt specific three-dimensional structures and functions. Related domains can be grouped by sequence similarity into domain families, which have a common evolutionary ancestry, and adopt similar structures and functions [4]. Domain families have been bioinformatically classified into databases such as CATH [5], the NCBI Conserved Domain Database [6], Interpro [7], and Pfam [8]. The Pfam v32.0 database contains a total of 17,929 domain families. These can be further classified into “clans”, sometimes referred to as domain superfamilies. Around 22 % (4049) of all domain families in Pfam v32.0 are defined as “domains of unknown function” or DUFs. DUFs can be recognized bioinformatically as families in sequenced genomes but have not been assigned a function. DUFs and other collections of uncharacterized protein families are a fascinating target for bioinformatic analysis, since many encode potentially novel biochemical activities and biological functions [9]. Identifying which DUFs are virulence factors and their potential mechanisms is an important task.

As an alternative to homology-based functional annotation methods, functional insights into protein families can be obtained by detecting statistical associations between families and various biological traits. Quantification of the relative abundance of a protein family across different environments can help provide insights into its functional context [10–16]. For example, Ellrott et al. [12], used an automated computational procedure to identify protein families specific to the human gut microbiome, and discovered 835 sequence families in metagenomic data. Subsequent experimental characterization of some of these protein families have revealed functions that are important for microbial physiology in the human gut [13]. Second, the presence/absence of a protein family may show a statistical association with a certain phenotype. For example, numerous studies have compared protein family abundance between pathogenic and non-pathogenic genomes to detect those that may play roles in virulence [17–19]. Levy et al. compared plant-associated bacterial genomes with non-plant-associated bacterial genomes and found 767 domains that were significantly associated with host-associated bacterial strains [20]. This led to the discovery of a gene family involved with inter-microbe competition in plant-associated environments.

In this work, we have applied several association-based methods to analyze 17,979 Pfam domain families in terms of their pathogen-association, taxonomic distribution, enrichment in the human gut, and other factors. Our analysis identifies hundreds of pathogen-associated domains, which include known and potentially novel candidate virulence factors for future characterization. We provide an online database (https://pathfams.uwaterloo.ca) to allow researchers to analyze their proteins of interest and explore our pathogen-associated domain families.

## Results and discussion

### Identification of pathogen-enriched domains

To identify domain families enriched in pathogens, we first constructed a dataset of 354 pathogen and 7897 non-pathogen bacterial proteomes (Data S1) based on the PATRIC database [21] and metadata from Dhillon et al. [22] (see Methods). The pathogens are associated with a wide-range of host species including not only humans, but also other animals, and plants [22]. Importantly, we acknowledge that a binary classification of “pathogen” versus “non-pathogen” is an oversimplification and may lead to biases based on taxon sampling and human annotation. We also appreciate issues related to pooling pathogens of multiple host species, which may reduce the host-specificity of our predictions and ability to make targeted predictions. Nevertheless, we hypothesized that comparative analysis of our pathogen vs. non-pathogen dataset could facilitate detection of protein domain families with an increased tendency to be involved in virulence-related interactions.

**Fig. 1:**
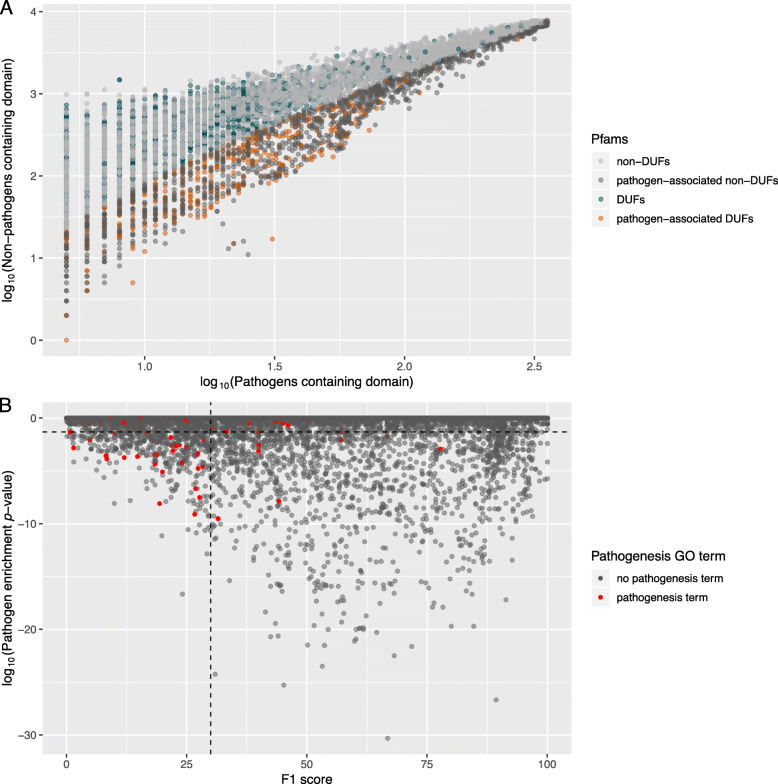
Scatterplots of Pfam domain pathogen-association. **a** Pfam domain presence in pathogen versus non-pathogen proteomes, with significant pathogen-associated patterns shown. Only domains present in > = 5 pathogens were included. **b** Trends in pathogenesis GO term annotation shown with respect to enrichment in pathogen proteomes and a measure of lineage specificity, the F1 score. The horizontal dotted line is at log_10_(0.05), showing the pathogen-association threshold. The vertical dotted line is at an F1 score of 30

For each Pfam domain, we calculated its statistical overrepresentation in pathogen proteomes using a hypergeometric test (see Fig. 1a). To account for proteome-specific duplications, which could bias the enrichment statistic, only binary presence/absence of the domain in a proteome was assessed. We identified 2007 significantly enriched (*p*_adj_ < 0.05) domains (including 517 DUFs), in the pathogenic set, out of 11,299 domains present in bacterial proteomes (Fig. 1a). Among pathogen-associated domains, DUFs were slightly enriched (1.16-fold, *p* = 4.4 × 10^− 4^). As expected, pathogenic lineages such as the Enterobacteriaceae had the highest frequency of pathogen-associated domains per proteome (Figure S1). Also, consistent with expectation, the GO term “pathogenesis” was significantly overrepresented in this set of Pfam domains (2.67-fold above background frequency in Pfam database, *p* = 1.50 × 10^− 6^). Among the top-scoring pathogen-associated Pfam families are numerous domains from known toxins and virulence factors (Table S1). For example, three of the four domains within the botulinum neurotoxin protein (Toxin_trans, Peptidase_M27, Toxin_R_Bind_N), a protein family previously thought to be restricted to *Clostridium* but recently demonstrated to be more broadly distributed [23–25], occurs in the top 20 pathogen-associated Pfam families (Table S1).

We also examined the degree to which pathogen-associated domain families represent known virulence factors based on existing virulence factor databases. 29 % of the proteins in the VFDB [2] and 32 % of the proteins in the Victors virulence factors database [26] contain at least one pathogen-enriched domain (Table S2). This coverage increased to 60 % when looking at proteins classified as “toxins” in the VFDB and 44 % for proteins with functions in “biofilm formation” (Table S2). This likely reflects the fact that current virulence factor databases include many non-specific proteins that contribute to but are not exclusively associated with virulence (e.g., common flagellar proteins, metabolic enzymes, and regulatory proteins).

### Incorporating taxonomic information enhances virulence-domain detection

Among the top-scoring predictions were many domains that were exclusive to one species or lineage of bacteria (e.g., *Mycoplasma*-specific domain families) (Table S1). To identify pathogen-associated domains that were more broadly distributed, we applied a lineage-specificity score (F1) metric [27] (see Methods) to filter taxonomically restricted domains. Combining pathogen-association and lineage-specificity scores together resulted in a significant increase in our ability to distinguish domains with the GO term “pathogenesis”. Particularly, domains not restricted to a particular lineage (F1 scores < 30) were 9-fold enriched in “pathogenesis” compared to domains with higher F1 scores (Fig. 1b). This is consistent with the idea that many pathogen-associated protein families (i.e. virulence factors) tend to undergo horizontal gene transfer and therefore may be less likely to exhibit high lineage-specificity [28]. This also illustrates the utility of combining lineage information and pathogen-association for virulence factor discovery. The top-scoring domains according to this combined criteria include IPT, DUF386, DUF2779. The IPT domain family encodes Isopentenyltransferases, which produce plant cytokinin, and are found both in plant pathogenic bacteria, plant-growth promoting bacteria, and plants [29]. The DUF386 domain family has been implicated in regulation of biofilm formation and sialic acid catabolism [30, 31]. DUF2779 is of unknown function but is a member of the Ribonuclease H (RNase_H) clan in Pfam.

A subset of virulence factors modulates or disrupts host function by “mimicry” of eukaryotic proteins [17, 32, 33]. To identify candidate virulence factors with eukaryotic-like domains, we intersected the set of bacterial pathogen-associated domains with domains identified as being most common in eukaryotes. The following “eukaryotic-like” domains were identified as overrepresented in pathogens: 7TM_GPCR_Sri, BRICHOS, Choline_kinase, Cystatin, Cytadhesin_P30, DIT1_PvcA, DNA_pol_B, DNA_pol_B_exo1, DUF1479, DUF1726, DUF1729, DUF3827, DUF762, Dynein_heavy, Ecl1, Ehrlichia_rpt, Elongin_A, EMP24_GP25L, Erp_C, F-box, F-box-like, GDA1_CD39, GNAT_acetyltr_2, Helicase_RecD, His_Phos_2, HMG_CoA_synt_C, HMG_CoA_synt_N, IES5, Latrotoxin_C, LMP, Methyltransf_10, MRG, MyTH4, Octapeptide, P_C10, P16-Arc, PAM2, PBC, PC4, Peptidase_M16_M, PhoLip_ATPase_C, Proteasom_PSMB, PTPlike_phytase, Rad33, RasGEF, SAT, Secs. 7, YMF19, and zf-Nse. Among the identified proteins are known examples of molecular mimicry by bacterial pathogens including the RalF virulence factor of *Legionella* which mimics host Sec7 guanine exchange factors (GEFs) (PF01369) [17]. Additional *Legionella* secreted effectors, such as a protein family containing a eukaryotic RAS-GEF domain (PF00617), are also included in this list. Other interesting predictions include the Latrotoxin_C domain (PF15658) found in the black widow spider insecticidal latrotoxin, but also present in *Wolbachia* species. Each of these cases implies an ancestral horizontal gene transfer event from a eukaryotic species to bacteria.

### Incorporating metagenomic information reveals pathogenic functions in the human gut microbiome

**Fig. 2:**
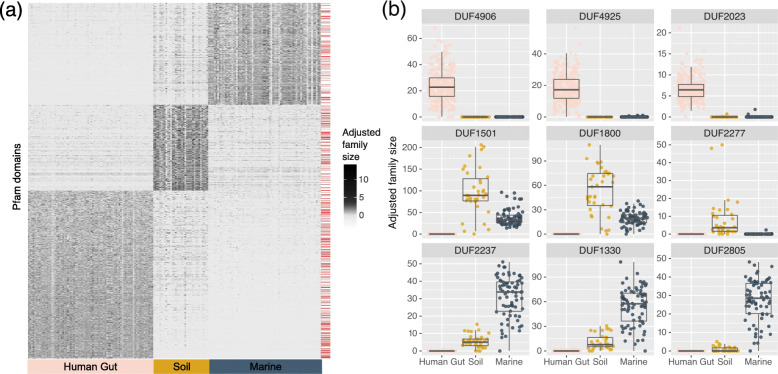
Detected Pfam families with strong environmental associations. **a** Abundance heatmap of Pfam families with significant environmental-specificity scores (*p*_adj_ < 1 × 10^− 15^). The adjusted family size was calculated as the logarithm of the normalized adjusted family size (base 10), scaled across the domain values. The red lines on the right-side of the plot denote DUF rows. **b** Selected DUF families with strong environment-specificity scores. Plotted are the per-sample distributions of normalized adjusted family size in three environments: human gut, marine, and soil

To further focus our predictions toward domains with potential human specificity, we examined domain family abundance in human gut metagenomes and compared it to that in soil and marine metagenomes (Data S2, S3). Using a stringent q-value threshold of 1 × 10^− 15^, we identified a set of 2061 domains with significant enrichment in the human gut. Additionally, we identified 1050 in soil, and 1246 in marine systems (see heatmap in Fig. 2a). Among the set of 4357 environment-associated families, 1056 (24.2 %) were DUFs. DUFs were most strongly enriched in human gut associated families (1.20 fold, *p* = 1.37 × 10^− 5^), followed by soil-associated families (1.13-fold, *p* = 0.016), and were underrepresented in marine-associated families (0.82 fold, *p* = 3.0 × 10^− 4^). Example DUFs with extreme environmental specificity are shown in Fig. 2b and top-scoring Pfam and DUF families are listed in Tables S3 and S4.

The top enriched GO terms for human-gut associated protein families included the phosphoenolpyruvate-dependent sugar phosphotransferase system, O-glycosyl hydrolase activity, and carbohydrate metabolic process (Table S5). Also, among the top human-gut enriched domains are domains with known roles in host adhesion/colonization and gut microbial metabolism (Table S5). Both characterized and uncharacterized (DUF) domains are found within this list. For example, DUF4906 (PF16249; ranked #1) appears to be a homolog of the fimbrial proteins Mfa2 (PF08842) and P_gingi_FimA (PF06321), known to be involved in cell adhesion. Fimbrillin_C (PF15495; ranked #11) is also associated with P_gingi_FimA. These domain families appear to be members of a broader superfamily of fimbrial proteins [13] in the human gut microbiome, and may be responsible for cell adhesion to the human gut epithelium. The identification of the carbohydrate-binding module CBM32 (PF18344; ranked in top 10) also makes sense from the perspective of microbial carbohydrate metabolism in the human gut. Finally, the identification of Maff2 (PF12750) within the top 10 domains also agrees with previous literature since this protein family is associated with tetracycline resistance cassettes that are extremely abundant in the human gut microbiome [34].

To identify candidate human gut virulence factors, we intersected the list of pathogen-associated domain families with domain families more enriched in the human gut microbiome than other environments (Data S3). The top 20 of these are listed in Table S6. We observed a striking enrichment of known virulence factors in these predictions with numerous DUF families interspersed (Table S6, Data S3). Families identified by this analysis include the LcrG family (PF07216), which encode a component of the *Yersinia* yop operon for secretion of virulence factors, BNR_3 (PF13859; bacterial neuraminidase repeat-like domain), HrpB7 (PF09486; type III secretion effector), Glyco_transf_52 (PF07922) which produces lipooligosaccharide (a pathogenicity determinant), the toxin family Thiol_cytolysin (PF01289), and the virulence factor Pertactin (PF03212). BNR_3 is a domain family that includes trans sialidases from the parasitic protist *Trypanosoma cruzi* (trans-sialidase; TcTS) and other *Trypanosoma* spp., as well as sialidases from human gut bacteria including *Prevotella* spp. and *Bacteroides* spp. Sialidases allow pathogenic bacteria and commensals to release free sialic acids in the gut as a nutrient source, and sialic acid catabolism has been demonstrated to promote the growth of gut pathogens (e.g., *E. coli*) and drive intestinal inflammation and dysbiosis [35].

DUFs within the list of pathogen-enriched and gut-enriched domains include DUF2492 (PF10678), DUF1430 (PF07242), and DUF3173 (PF11372). Based on InterPro descriptions for entries IPR019620 and IPR006541, DUF2492 appears to be a metal binding sulfatase and may play a role in sulfated mucin metabolism. DUF1430 appears to be a transporter and occurs in numerous pathogens including *C. difficile*, *Enterococcus*, and *S. pneumoniae*. DUF3173 (PF11372) is largely restricted to Firmicutes including numerous pathogens, and appears to be conserved near phage integrase genes. DUF families identified by this analysis are of particular relevance and we suggest should be prioritized for functional characterization in the context of human gut pathogenesis.

### PathFams: an online database for exploration of pathogen-associated domain families

**Fig. 3:**
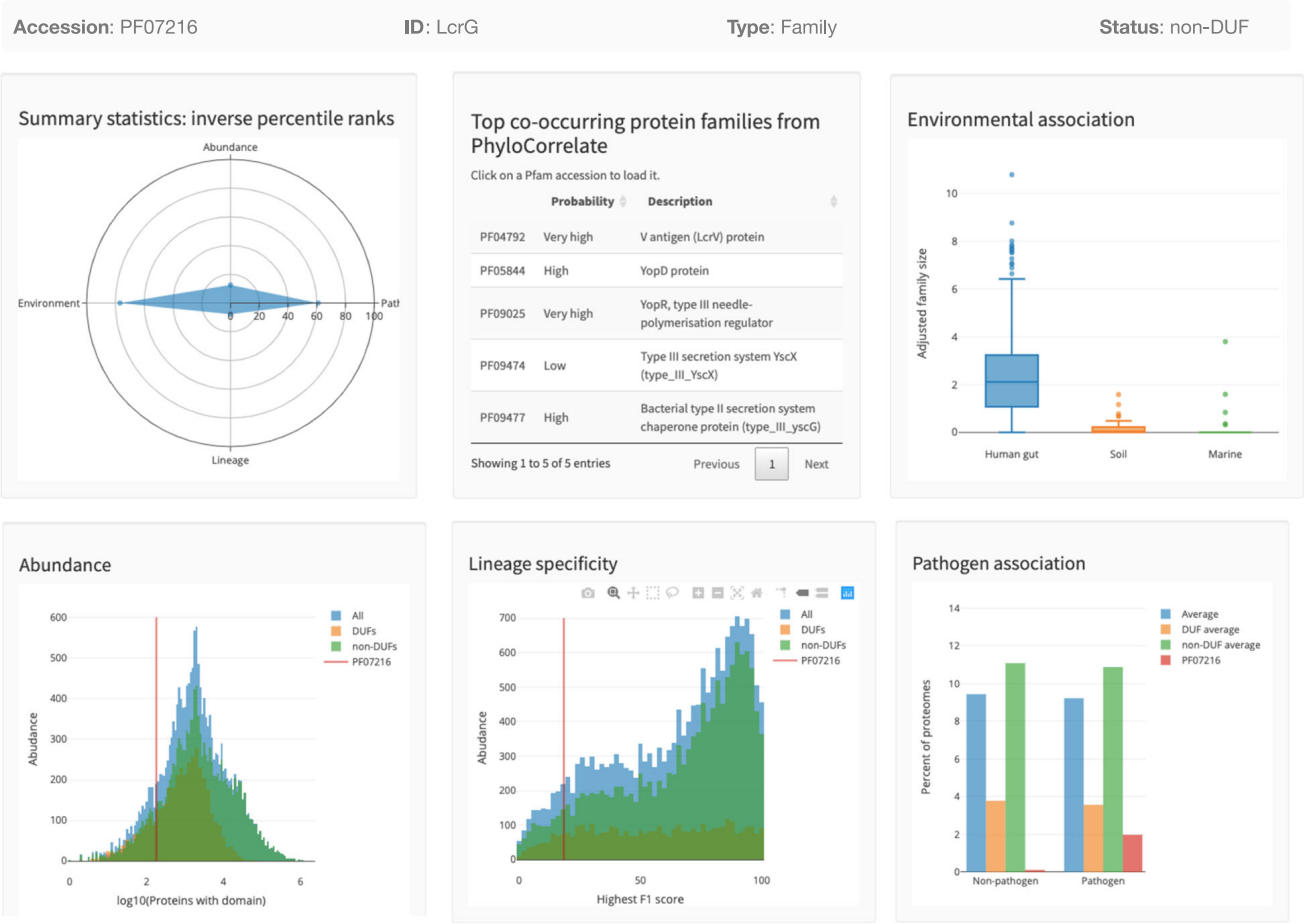
Screenshot of the domain info page from PathFams for the LcrG Pfam family (PF07216)

In order to provide these analyses to the community, we constructed an online database (pathfams.uwaterloo.ca) which facilitates interactive exploration of all Pfam domain families. Included are measures of abundance and taxonomic breadth, as well as indicators of structural determination feasibility (see Methods). As an example demonstrating the use of our database, Fig. 3 illustrates the PathFams page for Pfam family LcrG (PF07216) described earlier. A summary panel provides an overview of LcrG’s scores according to overall abundance, lineage-specificity, environmental association, and pathogen-association. This family is significantly enriched in the human gut metagenome, is significantly pathogen-associated, is non-lineage-specific and thus distributed across taxa, and is relatively low in abundance. PathFams also reports the top co-occurring Pfam domain families based on the PhyloCorrelate algorithm [36]. These include a variety of type III secretion system domains (#1 rank is LcrV), which is consistent with the known role of LcrG as a type III secretion system component [37].

**Fig. 4:**
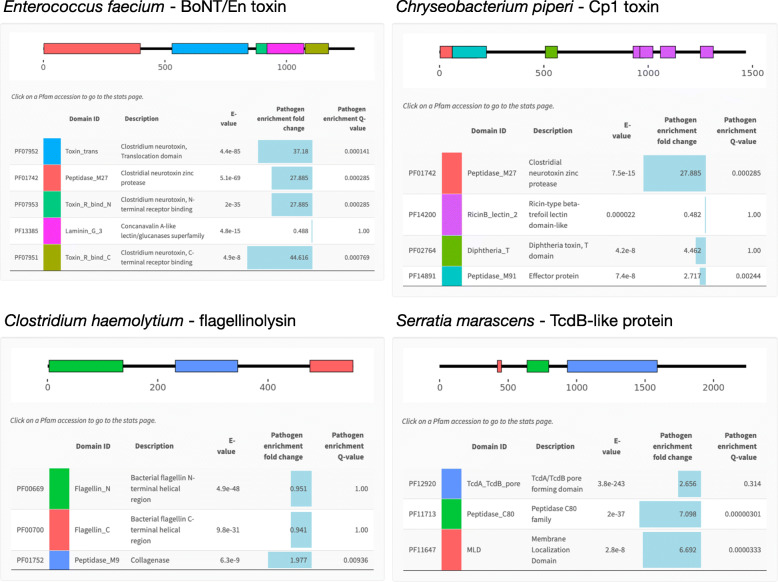
Detection of pathogen-associated domains and domain architectures for four example proteins by the online PathFams resource. Accession IDs are OTO22244.1 (*Enterococcus faecium* BoNT/En toxin), WP_034687872.1 (*Chryseobacterium piperi* Cp1 toxin), BAB87738.1 (*Clostridium haemolyticum* flagellinolysin), and OKB66574.1 (*Serratia marascens* hypothetical protein). Sensitive mode with an E-value cut-off of 1 × 10^− 7^ was used for all sequences except the *C. piperi* Cp1 toxin. For the *C. piperi* sequence, an E-value cut-off of 1 × 10^− 3^ was required to visualize the more divergent ricin domains

PathFams also allows the user to query the database with a protein sequence of interest. A submitted protein sequence will then be scanned against all Pfam models in the VirFam DB using either a “strict-mode” or “sensitive-mode”, and the predicted domain architectures will be visualized along with the pathogen-association scores for each identified domain (Fig. 4). Domains of interest can be explored further through links to their individual PathFams domain pages. Figure 4 shows example predictions for four recently discovered virulence factors. Despite occurring outside of the *Clostridium* taxonomic lineage, a botulinum neurotoxin (BoNT) like protein was identified in the gut commensal organism *Enterococcus faecium* [25]. The PathFams prediction for this protein identifies all four domains found in BoNT and also correctly reports them as being significantly pathogen-associated. An additional BoNT-like protein called Cp1 was also recently discovered in *Chryseobacterium piperi*, but this protein appears to possess structural differences from BoNTs and displays cytotoxic versus neurotoxic function [16]. The PathFams prediction for Cp1 is consistent with this, since it is predicted to contain a BoNT-like peptidase_M27 N-terminal domain that is highly pathogen-associated, a diphtheria-like translocation domain which is also > 4-fold enriched in pathogens, and a C-terminus composed of ricin-like repeats that are not pathogen-associated and occur more broadly. As a third example, a novel variant of bacterial flagellin called flagellinolysin was recently discovered in the animal pathogen *Clostridium haemolyticum* as well as in diverse bacterial taxa [38]. Flagellinolysin is unique from other flagellins by possessing a central zinc-metalloproteinase domain, which in *C. haemolyticum* provides flagellar filaments with proteolytic activity against extracellular host substrates [38]. Consistent with this, the PathFams prediction for flagellinolysin reveals a domain architecture including standard N- and C-terminal flagellin with no pathogen-association, but also detects a central collagenase-like (M9) protease domain that is predicted to be significantly (> 2-fold) enriched in pathogens. As a final example, recent work has shown that the large clostridial toxins from *Clostridium difficile* (TcdA and TcdB) are highly abundant outside of the *Clostridium difficile* lineage [39]. These TcdA/B-like proteins represent candidate virulence factors. Shown in Fig. 4 is the PathFams prediction for one of these proteins from the opportunistic pathogen *Serratia marascens*, which is currently annotated in the NCBI database as a “hypothetical protein” (NCBI accession WP_073532240.1). Three domains common to TcdA and TcdB are predicted within this protein, all of which are pathogen-associated. As *S. marascens* is an insect pathogen and the top homologs of this protein according to BLAST occur in insect pathogenic *Photorhabdus* spp., we suggest that protein is likely an insecticidal toxin. We anticipate that, similar to these cases, other candidate virulence factors may be identified using PathFams tool by assessing domain architectures of uncharacterized proteins and identifying those that show significant pathogen associations.

## Conclusions

In this work, we analyzed all 17,929 protein domain families in the Pfam v32.0 database in order to rank them based on several biological criteria. We were able to identify significant lineage, pathogen, and/or environment associations for 1675 out of 4049 (41 %) of all DUFs. These associations provide a biological context from which uncharacterized domain families (DUFs) can be prioritized for future virulence factor studies. In addition, by combining different scores, it was possible to identify Pfam families with specific phenotypic or functional associations, such as candidate virulence factors in the human gut microbiome, as well as candidates predicted to be feasible for structure determination. PathFams provides a clear and interactive way to explore this data, allowing users to assess the virulence factor potential of a domain family and/or submitted protein. Future work will update and expand our computational analysis of the Pfam database to include new metadata and phenotypic associations, and new domain families as they continue to be uncovered through ongoing sequencing efforts.

## Methods

### Pathogen association

354 proteomes in Pfam v32.0 were designated as bacterial pathogens (Data S1) based on PATRIC (https://www.patricbrc.org) [21] bacterial pathogens with metadata relating them to disease and a manually curated set of pathogens from Dhillon et al. [22]. Specifically, genera containing “Pathogens” were identified under the “Organisms” list within PATRIC, and genomes with disease metadata were selected. These genomes as well as the Dhillon et al. [22] set were then mapped to Pfam proteomes based on species names. All other Pfam proteomes that were not defined as a pathogen by PATRIC or Dhillon et al. [22] were included in the “non-pathogen” category.

Enriched pathogenic domains were detected with the hypergeometric test (phyper in R) based on the number of pathogenic proteomes in Pfam where the domain is present, compared to non-pathogenic bacterial proteomes in Pfam where the domain is present. The *p*-values were FDR corrected with p.adjust using the Benjamini-Hochberg model. The enrichment of DUFs in pathogen-associated domains was calculated in the same way as with the environment-associated domain set. For this paper, Pfam domains called UPF (uncharacterized protein family) were also treated as DUFs. The frequency of the pathogenesis GO term in domains identified as pathogen-associated and other Pfam domains present in bacterial proteomes were compared with the hypergeometric test (phyper in R). Eukaryotic-like domains in bacterial pathogens were identified as being most common in eukaryotic proteomes as well as pathogen-associated (*p*-value < 0.05) or with hits in bacterial pathogens but without hits in non-pathogen proteomes. We expanded past the pathogen-associated domain set in this case, to capture domains present in a low number of proteomes (which meant they weren’t statistically significant) that seemed like promising “mimicry” candidates.

In order to examine domain prevalence in virulence factor databases, the Victors [26] protein sequences and the protein sequences from VFDB’s full dataset [2] were downloaded from their websites (http://www.phidias.us/victors/downloads/gen_downloads_protein.php and http://www.mgc.ac.cn/VFs/Down/VFDB_setB_pro.fas.gz, respectively on Jul. 28, 2021). These proteins were annotated with PfamScan (version updated on Feb. 28, 2017; default settings) using HMMER3 v.3.1b2 [40] against the Pfam database v32.0. The “Intra-genera VFs comparison tables” (http://www.mgc.ac.cn/VFs/Down/Comparative_tables_from_VFDB.tar.gz) were used to extract the virulence factor category (e.g. “toxin” or “adherence”) to gene mapping. The prevalence of proteins with at least one pathogen-enriched domain (as determined previously) was calculated in the two different virulence factor databases, and within various virulence factor categories from VFDB.

### Lineage association

The taxonomy ID and taxonomic lineage of proteomes with Pfam domain matches were extracted, respectively, from PfamA_ncbi.txt.gz and taxonomy.txt.gz at Pfam’s ftp server (Pfam v.32.0; retrieved Oct. 16, 2018). We calculated the sensitivity and precision of the Pfam domain distribution across the NCBI taxonomy system using these taxonomy ids and taxonomic lineages. The total number of proteomes within any one taxonomic group is based on the taxonomy ids in the PfamA_ncbi.txt file. These scores were calculated for the most common taxon (presence/absence counts of a domain hit per proteome) in each domain family at the Superkingdom, Kingdom, Phylum, Class, Order, Family, and Genus taxonomic levels. The best taxonomic level to describe a domain’s lineage specificity was chosen based on the F1 score: 2*(sensitivity*precision)/(sensitivity + precision). In the case of a tie between taxonomic levels, the higher level in the taxonomic hierarchy (i.e. Superkingdom) was given preference. If the majority of proteomes that the domain was present in did not have any classification at a certain taxonomic level, this taxonomic level would not be considered for “best taxonomic level.” The enrichment of DUFs in extreme lineage-specific cases was determined in the same way as with the environmental-associated domain set.

### Environmental association

Metagenomic assemblies and raw reads were taken from public repositories (Data S2). No samples smaller than 1,000,000 bp were used. The raw reads from the human gut studies [41, 42] were processed and assembled with the following procedure. Any read that aligned to the human genome (GCA_000306695.2) with Bowtie 2 (v2.2.9) [43] default settings was removed (along with its pair). Quality trimming was performed by sickle v1.33. The reads were assembled with Megahit v1.0.6-3-gfb1e59b [44] with default settings. The raw reads from the Global Ocean Sampling study [45] were not assembled as the reads, which were sequenced with a modified form of Sanger sequencing, were already quite long. FragGeneScan v1.30 [46] was used to detect coding sequences (CDSs) in the samples. To remove any putatively spurious CDSs, any CDS with greater than 40 % repetitive sequence, detected by segmasker from the BLAST package v2.2.28+, was removed. Annotation with PfamScan (version updated on Feb. 28, 2017; using default settings) using HMMER3 v.3.1b2 [40] against the Pfam database v32.0 with a threshold of 1 × 10^− 3^ was performed on the remaining sequences. The annotated region of each metagenomic sequence (aligned with a Pfam domain) was clustered with CD-HIT v4.6.8 [47] to 99 % similarity for each sample within each set of domain matches. This removed redundant domain matches to give a measure of adjusted family size of the domain families for each sample. To normalize to sample size, the adjusted family member count was divided by the number of base pairs in the assembly and multiplied by 1,000,000. A ratio of samples across each human gut study analyzed was chosen to maximize regional diversity while making the sample size in each environment more comparable. We used all 14 healthy samples from the Spanish cohort [48], and then randomly selected 34, 16, and 16 healthy samples from the Danish cohort [48], the Chinese cohort originating from Peking University Shenzhen Hospital, Shenzhen Second People’s Hospital and Medical Research Center of Guangdong General Hospital [41], and the Chinese cohort originating from the First Affiliated Hospital of Zhejiang University [42], respectively (see Data S2). However, in per-domain figures all human gut samples have been added back in for visual comparison. Domains not present in greater than 95 % of the selected samples were excluded. Domains where at least one environment (soil, marine or human gut) showed significant differences based on the normalized adjusted family size were determined with the Kruskal-Wallis test. *p*-values were adjusted with p.adjust using the Benjamini-Hochberg model. The logarithm of the normalized adjusted family size (base 10) and the subsequent scaling across the domain hits (scale) was done in R v3.3.3 for the heatmap. Enrichment of DUFs in the environment-associated domain sets compared to the background frequency of DUFs in Pfam was tested using the binomial test (pbinom in R). To determine GO term enrichment within the environment-associated domain sets, a Pfam to GO term map was retrieved from http://geneontology.org/external2go/Pfam2go (last updated February 12, 2019). The frequency of GO terms in domains associated with one of the three environments (soil, marine and human gut) and the frequency of GO terms corresponding to other Pfam domains present in at least 5 % of the selected samples were compared with the hypergeometric test (phyper in R), with *p*-values again adjusted with the Benjamini-Hochberg model.

### Abundance and taxonomic breadth

The NCBI sequence database domain alignments were sourced from.www.ftp.ebi.ac.uk/pub/databases/Pfam/current_release/Pfam-A.full.ncbi (Pfam v.32.0; retrieved Feb. 9, 2019). The proteins that were aligned to Pfam domains and the total number of hits were taken from this file. An environmental average of the normalized adjusted family size for each domain (see *Environmental association* section of Methods) present in at least 5 % of the selected samples used to determine environment-association was calculated for Data S3. For taxonomic breadth, the proteomes with domain hits, their taxonomy ids and taxonomic lineages were used (see the *Lineage specificity* section in Methods). The percentage of species where each domain is present, and the corresponding percentage for the Genus, Family, Order, Class, Phylum, Kingdom and Superkingdom taxonomic levels are included in Data S3. Spearman rank correlations between the different abundance measures (percentage of species, environmental average, and protein hits in NCBI) were calculated with the corr function in R v3.3.3. Using the above data, we calculated three abundance metrics: N_NCBI_, the number of protein family members in the NCBI sequence database; N_species_, the percentage of species containing the domain family in the Pfam proteome collection; and N_meta_, the number of non-redundant matches in a diverse dataset of metagenomes.

### Additional filters

All data was taken from Pfam v.32.0 (files retrieved on Oct.16, 2018). A list of Pfam families with PDB structures was taken from.

www.ftp.ebi.ac.uk/pub/databases/Pfam/current_release/database_files/pdb_PfamA_reg.txt. Domain architectures were sourced from.

www.ftp.ebi.ac.uk/pub/databases/Pfam/current_release/database_files/architecture.txt.

Predicted transmembrane and disordered regions in sequences with Pfam domain alignments were retrieved from.

www.ftp.ebi.ac.uk/pub/databases/Pfam/current_release/database_files/other_reg.txt. Overlap of predicted transmembrane or disordered regions with an annotated domain was evaluated by comparing to www.ftp.ebi.ac.uk/pub/databases/Pfam/current_release/Pfam-A.regions.uniprot.tsv. The standard deviation for domain family percentage disorder was calculated using std from the NumPy package v1.16.1. Domains that were prioritized for structural feasibility had no representatives in the PDB, an average across the domain family members of less than 10 % of the domain sequence predicted to be disordered, less than 10 % of their members with a predicted transmembrane region (anywhere along the protein), and less than 10 % of their members with transmembrane-domain overlap.

## Supplementary Information

**Additional file 1.**

**Additional file 2.**

## Data Availability

The dataset supporting the conclusions of this article is included within the article’s additional files as Data S3.
