# Supplementary material for: PathFams: statistical detection of pathogen-associated protein domains
Source: BMC Genomics. 2021 Sep 14;22:663. doi: 10.1186/s12864-021-07982-8 (PMC8442362; doi:10.1186/s12864-021-07982-8)
Supplement: Supplementary file 2 — Additional file 2. [file 12864_2021_7982_MOESM2_ESM.pdf]

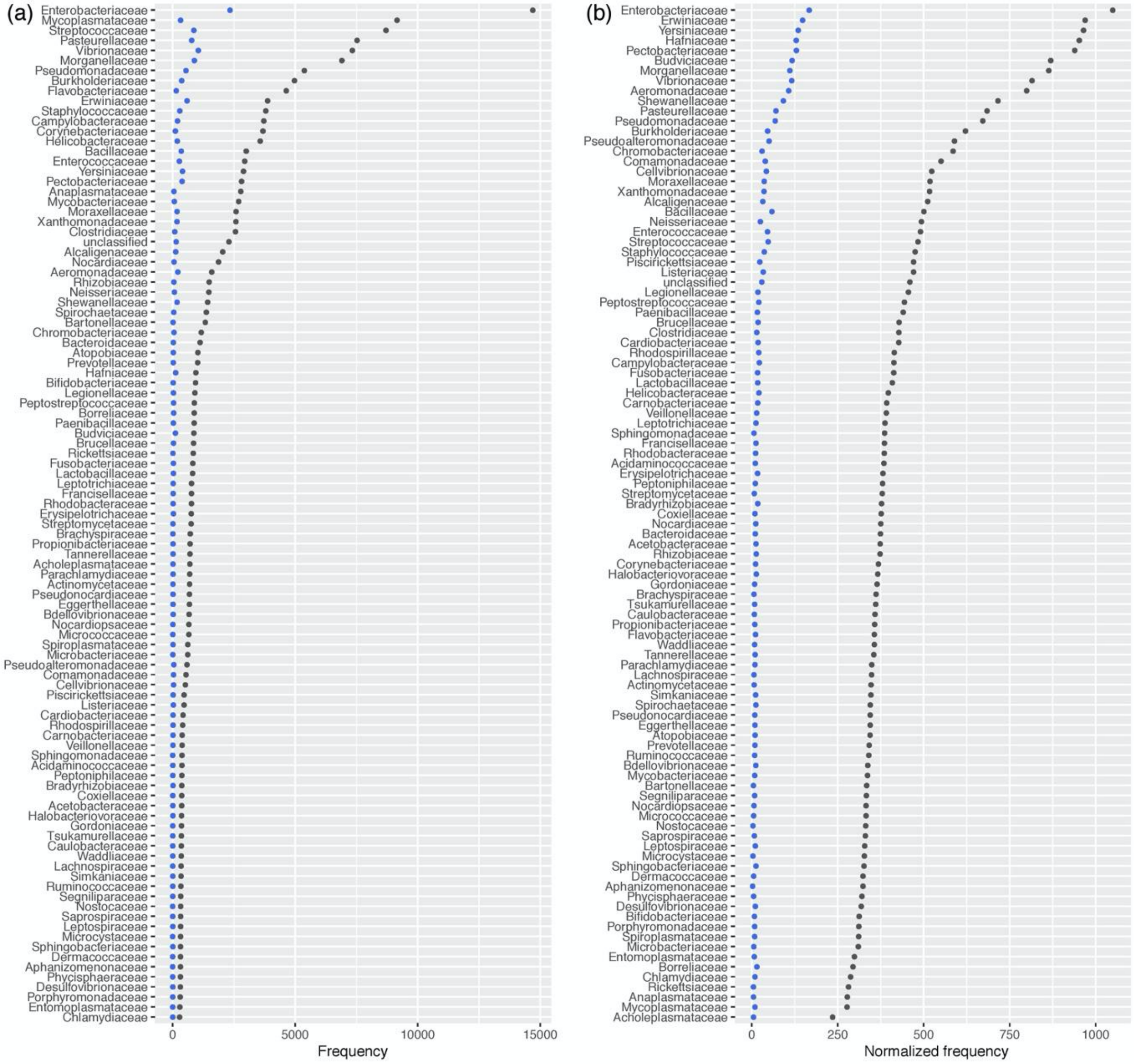

Figure S1: Distribution of family level taxonomic groups within the pathogen-enriched domain set. Total instances (a) and instance rate (b) normalized by number of pathogen proteomes in that taxonomic family. Each plot is reordered based on frequency. DUFs are in blue.

Table S1: Top 20 pathogen-associated domains. Families were ranked by fold change and are present in at least 5 pathogens. Proteomes with domain include all proteomes in the Pfam proteome collection (not just bacteria) and so may be larger than the sum of the Pathogens and Background (bacterial) columns.

	Pathogens	Background (bacterial)	P_{adj}	Fold change (bacterial)	Proteomes with domain
**DUF1410**	5	1	0	111.54	6
**DUF1600**	5	2	0	55.77	7
**DUF5378**	5	2	0	55.77	7
**Leader_Trp**	5	2	0	55.77	7
**MFS_Mycoplasma**	25	11	0	50.7	36
**DUF31**	31	17	0	40.68	48
**DUF5385**	9	5	0	40.15	14
**Lambda_Kil**	5	3	0	37.18	49
**Staphopain_pro**	5	3	0	37.18	8
**Toxin_trans**	5	3	0	37.18	9
**Lipoprotein_X**	21	13	0	36.04	34
**DUF2618**	6	4	0	33.46	10
**DUF2684**	6	4	0	33.46	10
**FinO_N**	6	4	0	33.46	11
**Mycoplasma_p37**	24	16	0	33.46	40
**DUF2714**	22	15	0	32.72	37
**Lipoprotein_10**	22	15	0	32.72	37
**Strep_SA_rep**	7	5	0	31.23	15
**Peptidase_M27**	5	4	0	27.88	10
**Toxin_R_bind_N**	5	4	0	27.88	13

Table S2: Pathogen-enriched domain coverage of virulence factor databases. The number of proteins with at least one pathogen-enriched domain in the different virulence factor databases, as well as within certain virulence factor categories from VFDB. The proportion of this pathogen-enriched domain coverage is shown for proteins in the entire dataset and for only proteins in the dataset that had a domain match to Pfam v32.0.

	Proteins with a pathogen-enriched domain	Out of proteins with a Pfam domain (%)	Out of total proteins (%)
**Victors**	1579	34	32
**VFDB (full dataset)**	8341	32	29
**Toxins**	470	63	60
**Biofilm formation**	47	50	44
**Secretion system**	2027	33	28
**Iron uptake**	622	31	31
**Adherence**	1417	27	26
**Enzyme**	100	24	23
**Invasion**	526	21	21
**Motility**	381	18	18
**Regulation**	103	16	16
**Immune evasion**	64	13	13

Table S3: Top 5 environment-associated domains from soil, marine, and human gut metagenomes. Shown in the table are the normalized average adjusted family sizes of each domain family in each environment. The list is ranked by the *p*-value and then by the normalized average adjusted family size of the environment the domain family is associated with.

	P_{adj}	Soil	Marine	Human Gut
**Soil - associated**				
**YceI**	3.27×10^{-33}	50.96	16.56	0.60
**Virul_fac_BrkB**	8.95×10^{-33}	113.75	13.41	33.69
**zf-HC2**	9.67×10^{-33}	72.42	1.29	17.35
**DUF1501**	1.10×10^{-32}	97.85	38.51	0.00
**GerE**	1.22×10^{-32}	242.00	21.89	109.79
**Marine - associated**				
**T4_neck-protein**	2.13×10^{-33}	0.07	31.79	0.03
**UvsY**	2.13×10^{-33}	0.07	22.92	0.01
**Gp5_OB**	2.13×10^{-33}	0.04	17.31	0.00
**Phage-Gp8**	2.14×10^{-33}	0.03	43.29	0.01
**DUF2237**	2.14×10^{-33}	5.61	30.92	0.01
**Human gut - associated**				
**DUF4906**	2.13×10^{-33}	0.00	0.00	23.80
**DUF4925**	2.13×10^{-33}	0.00	0.04	17.62
**LPD16**	2.13×10^{-33}	0.05	0.00	14.15
**Cys_rich_VLP**	2.13×10^{-33}	0.01	0.00	10.28
**Lipocalin_8**	2.13×10^{-33}	0.06	0.02	7.87

Table S4: Top 5 environment-associated DUFs from the soil, marine, and human gut. Shown in the table are the normalized average adjusted family sizes of each domain family in each environment. The list is ranked by the *p*-value and then by the normalized average adjusted family size of the environment the domain family is associated with.

	Padj	Soil	Marine	Human Gut
**Soil - associated**				
**DUF1501**	1.10×10^{-32}	97.85	38.51	0.00
**DUF1800**	1.35×10^{-32}	53.35	19.41	0.00
**DUF2277**	1.52×10^{-32}	7.99	0.04	0.00
**DUF2382**	1.94×10^{-32}	24.83	0.00	0.01
**DUF488**	2.42×10^{-32}	29.67	0.96	9.72
**Marine - associated**				
**DUF2237**	2.14×10^{-33}	5.61	30.92	0.01
**DUF1330**	4.54×10^{-33}	10.95	54.19	0.08
**DUF2805**	7.59×10^{-33}	0.87	27.34	0.00
**DUF4815**	7.90×10^{-33}	0.55	95.72	0.09
**DUF2061**	1.08×10^{-32}	0.73	15.74	0.01
**Human gut - associated**				
**DUF4906**	2.13×10^{-33}	0.00	0.00	23.80
**DUF4925**	2.13×10^{-33}	0.00	0.04	17.62
**DUF2023**	2.14×10^{-33}	0.02	0.03	6.55
**DUF4317**	2.91×10^{-33}	0.28	0.05	25.03
**DUF5119**	2.91×10^{-33}	0.07	0.03	16.50

Table S5: GO term enrichment in environment-associated domain sets. Fold change is within domains with a Pfam annotation in the environmental samples.

	Environment-associated domains with GO term	Non-environment-associated domains with GO term	Fold change	Padj
**Soil - associated**				
**transposase activity**	9	2	50.4	$9.85x10^{-6}$
**transposition, DNA-mediated**	11	3	41.1	$7.95x10^{-7}$
**heme binding**	9	15	6.72	$4.38x10^{-2}$
**oxidation-reduction process**	43	184	2.62	$1.36x10^{-4}$
**Marine - associated**				
**cytochrome complex assembly**	6	0	Inf	$3.08x10^{-4}$
**photosystem II reaction center**	6	1	55.7	$1.26x10^{-3}$
**photosynthesis, light reaction**	5	1	46.4	$1.02x10^{-2}$
**flavin adenine dinucleotide binding**	9	3	27.8	$6.55x10^{-5}$
**photosystem I**	8	3	24.7	$3.19x10^{-4}$
**oxidoreductase activity, acting on the CH-CH group of donors**	5	2	23.2	$2.63x10^{-2}$
**tricarboxylic acid cycle**	5	2	23.2	$2.63x10^{-2}$
**nickel cation binding**	5	2	23.2	$2.63x10^{-2}$
**photosynthesis**	25	16	14.5	$1.15x10^{-12}$
**photosystem II**	11	8	12.8	$1.10x10^{-4}$
**Human gut - associated**				
**mismatch repair**	8	0	Inf	$3.81x10^{-4}$
**mismatched DNA binding**	6	0	Inf	$8.88x10^{-3}$
**spore germination**	5	0	Inf	$3.95x10^{-2}$
**phosphoenolpyruvate-dependent sugar phosphotransferase system**	11	4	14.3	$8.53x10^{-4}$
**cobalamin biosynthetic process**	9	4	11.7	$1.17x10^{-2}$
**hydrolase activity, hydrolyzing O-glycosyl compounds**	22	16	7.17	$6.50x10^{-6}$
**carbohydrate metabolic process**	39	35	5.81	$9.65x10^{-10}$

Table S6: Top 20 pathogen-associated Pfam families that are also enriched in the human gut microbiome. Families are ranked by fold change in pathogen proteomes. N = # proteomes with domain, f_P = # bacterial pathogen proteomes with domain, f_{np} = # non-pathogen proteomes (bacterial) with domain.

	N	f_P	f_{np}	P_{adj}	Fold change in pathogens	Human gut environment-association (P_{adj})
**LcrG**	16	7	9	$1.60x10^{-7}$	17.35	$1.37x10^{-21}$
**DUF4948**	13	3	10	$4.30x10^{-2}$	6.69	$7.23x10^{-27}$
**Mac-1**	47	10	36	$2.67x10^{-5}$	6.20	$4.18x10^{-16}$
**Gp58**	58	6	24	$3.62x10^{-3}$	5.58	$2.79x10^{-18}$
**BNR_3**	47	7	30	$2.05x10^{-3}$	5.21	$1.75x10^{-20}$
**zinc-ribbons_6**	175	32	142	$5.02x10^{-13}$	5.03	$4.02x10^{-21}$
**HrpB7**	44	8	36	$1.14x10^{-3}$	4.96	$1.68x10^{-23}$
**Glyco_transf_52**	120	21	99	$2.94x10^{-8}$	4.73	$1.23x10^{-21}$
**DUF2492**	202	35	166	$2.74x10^{-13}$	4.70	$2.27x10^{-25}$
**HDC**	43	7	34	$4.33x10^{-3}$	4.59	$7.11x10^{-27}$
**Thiol_cytolysin**	187	30	153	$1.20x10^{-10}$	4.37	$5.38x10^{-21}$
**Glyco_hydro_98C**	32	5	27	$3.25x10^{-2}$	4.13	$9.11x10^{-19}$
**HU-DNA_bdg**	58	9	49	$1.97x10^{-3}$	4.10	$2.27x10^{-23}$
**PagP**	245	37	203	$5.23x10^{-12}$	4.07	$1.22x10^{-16}$
**CBM32**	40	6	34	$2.01x10^{-2}$	3.94	$2.13x10^{-33}$
**Pertactin**	316	45	266	$2.79x10^{-13}$	3.77	$1.23x10^{-17}$
**DUF1430**	98	14	84	$1.70x10^{-4}$	3.72	$3.62x10^{-21}$
**DUF3173**	112	16	96	$4.76x10^{-5}$	3.72	$1.76x10^{-18}$
**Glyco_hydro_98M**	42	6	36	$2.59x10^{-2}$	3.72	$1.46x10^{-18}$
**MuF_C**	121	15	91	$1.02x10^{-4}$	3.68	$1.86x10^{-19}$
